# β‐Galactosidase deficiency in the GLB1 spectrum of lysosomal storage disease can present with severe muscle weakness and atrophy

**DOI:** 10.1002/jmd2.12324

**Published:** 2022-09-06

**Authors:** Jonas Jalili Pedersen, Morten Duno, Flemming Wibrand, Christian Hammer, Thomas Krag, John Vissing

**Affiliations:** ^1^ Copenhagen Neuromuscular Center, Department of Neurology Rigshospitalet, University Hospital Copenhagen Copenhagen Denmark; ^2^ Molecular Genetic Laboratory, Department of Clinical Genetics Rigshospitalet, University Hospital Copenhagen Copenhagen Denmark; ^3^ Metabolic Laboratory, Department of Clinical Genetics Rigshospitalet, University Hospital Copenhagen Copenhagen Denmark; ^4^ Diagnostic Center, Department of Radiology Rigshospitalet, University Hospital Copenhagen Copenhagen Denmark

**Keywords:** fat replacement, GLB1‐related disorders, metabolic neuromuscular disorder, Morquio B, muscle wasting, myopathy, β‐galactosidase deficiency

## Abstract

Deficiency of the enzyme β‐galactosidase due to variants in the *GLB1*‐gene is associated with metabolic disorders: Morquio B and GM1‐gangliosidosis. Here, we report a case compound heterozygous for variants in the *GLB1*‐gene and a severe muscular phenotype. Full body T1‐w MRI was conducted for muscular involvement. Biopsy was stained with hematoxylin and eosin for histopathological evaluation. EDTA blood‐sample was subjected to whole exome sequencing. Metabolic analysis included residual enzyme activity and evaluation urinary substrate secretion. Additionally, electroneurography, echocardiography, forced volume capacity and biochemistry were evaluated. Examination showed severe proximal weakness (MRC: hip flexion 2, hip extension 2, and shoulder rotation 2), Gower's sign, no extrapyramidal symptoms and normal creatine kinase levels. MRI showed severe muscle wasting of the thigh and shoulder girdle. Muscle biopsy showed mild myopathic changes. β‐galactosidase activity was reduced to 28%–34%. Urinary glycosaminoglycan was elevated by 5.9–8.6 mg/mmol (ref.:0–5.1 mg/mmol). Electrophoresis indicated excess keratan sulfate. Exome sequencing revealed two missense variants in the *GLB1* gene. Clinical features, genetic testing and laboratory findings indicate a case of β‐galactosidase‐deficiency with a muscular phenotype.

## 1 INTRODUCTION

Pathogenic variants in the *GLB1* gene may lead to reduced activity of the lysosomal hydrolase, β‐galactosidase, and result in lysosomal storage disorders: GM1 gangliosidosis (OMIM #230500) and mucopolysaccharidosis type IVB, also known as Morquio B Disease (MBD, OMIM #253010). Both are rare autosomal, recessively inherited with prevalence between 1:100.000–300.000 (GM1‐ganglioside) and 1:250.000–1:1.000.000 (MBD).^1^ β‐galactosidase facilitates degradation of structurally unrelated molecules such as ganglioside, oligosaccharide, and glycosaminoglycan (GAG) in the lysosomes, which otherwise accumulate and cause disruption in case of β‐galactosidase deficiency.^2^ MBD is characterized by involvement of the bones and an absence of neurological manifestations. Symptoms include growth impairment, kyphoscoliosis, coxa/genu valga, chronic pain, and mobility issues. Other systemic features include corneal clouding, hearing impairment, respiratory compromise, hepatosplenomegaly, and cardiac dysfunction.^3^, ^4^ Laboratory findings include oligosaccharide and GAG (specifically keratan sulfate) secretion in urine.^5^ The second phenotype, GM1‐ganglioside, is characterized by its neurodegenerative involvement, as accumulation of GM1‐ganglioside leads to apoptosis of neurons in the CNS.^6^ Genetic findings of pathogenic variants in the *GLB1* gene and/or low residual activity levels of β‐galactosidase support the diagnosis of both disorders.^7^ In both disorders, disease severity attenuates with later age of onset, and GM1‐ganglioside especially can be fatal if present at birth.^1^ Here, we report a case compound heterozygous for variants in the *GLB1* gene with some MBD features and a severe neuromuscular phenotype of muscle wasting and weakness.

## 2 CASE STORY

**FIGURE 1:**
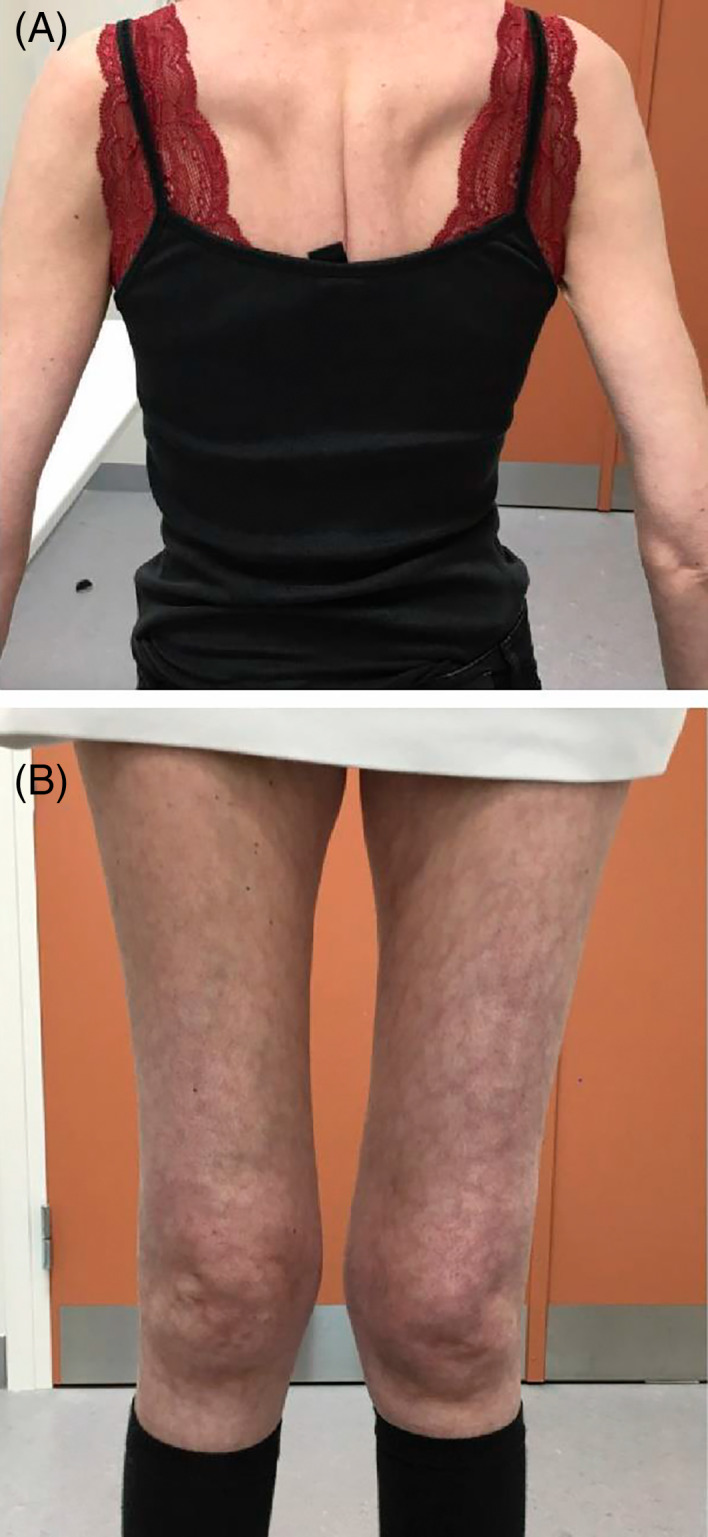
Clinical pictures of the patients back and thigs. (A) Shows scapulae alatae. (B) shows pronounced atrophy of the thigh muscles

The index case is a now 41‐year‐old woman who was referred to our clinic at the age of 21 years with muscle weakness and difficulties in walking. There was no history of neurological disease in the family. Her mother died from pulmonic emboli at 63 years of age, and the father is still alive at age 71 years. Postnatal development and early motor milestones were normal. At the age of five, her parents noticed that she was slow at running compared to other kids. In her early teens, the patient did not experience significant problems and enjoyed bicycling and roller‐skating. She finished elementary school and continued to do a 2‐years high school degree of business education. At age 19 years, she started noticing difficulties walking stairs. The following years, there was a slow but steady progression of symptoms with increasing difficulties in performing activities of daily life. By age 30 years, she started visiting a pulmonary outpatient clinic due to respiratory compromise. In her mid‐30s, her muscle weakness further progressed and she had difficulties rising up from lying down. At this point, she also began experiencing frequent falls when walking due to mobility issues. The patient reported significant pain around the hips, knee, and forefoot, leading to frequent visits to an orthopedic clinic. She struggled with episodes of tinnitus and was described with signs of cataract at the age of 30. Presently, the patient works 3–4 h daily with a desktop job, refrains from walking stairs or hilly terrain and is dependent on home care. On exam, she presents with muscular atrophy most prominent in thigh and shoulder girdle (BMI 15.5), scapulae alatae and severe atrophy of muscles of the thigh (Figure 1A). She has Gower sign when rising from a chair, and walks with a hyperlordotic waddling gait. There is significant genu valgum when standing (Figure 1B). She has no extrapyramidal symptoms and has universal areflexia of deep tendon reflexes. There is severe proximal weakness (MRC: hip flexion 2, hip extension 2, shoulder rotation 2, shoulder abduction 4, knee extension 2, and knee flexion 3). Currently, it impossible for her to rise from lying down without support.

### 2.1 Genetic testing

DNA was isolated from a standard EDTA blood‐sample and subjected to whole exome sequencing, screening for potential pathogenic variants related to the phenotype. The patient was found to be compound heterozygous for two missense variants c.113G>A, (p. Arg38Gln) and c.245C>T, (p. Thr82Met) in the *GLB1* gene (NM_000404.3). The latter (c.245C>T) is known to be associated with late onset GLB1‐disorders.^3^, ^8^ The c.113G>A variant has not previously been associated with a pathological phenotype, and is known with a prevalence of 5/128.000 (gnomAD v.2.21, Europeans). In silico analysis did not predict a strong pathogenicity, although the arginine at position 38 is moderately conserved. The father only carries one of the two variants, suggesting that the two *GLB1* variants are biallelic in the proband.

### 2.2 Imaging

**FIGURE 2:**
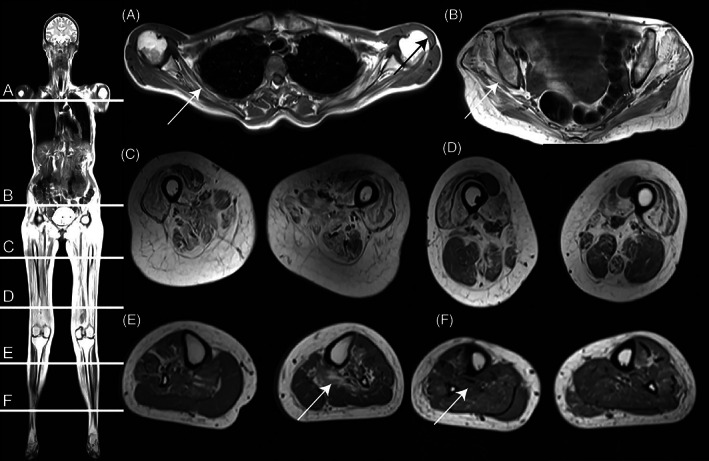
Full body localizer (left) and T1‐weighted cross‐sectional image (A–F) shows significant fat replacement with a proximal to distal gradient affection. (A) Shoulder girdle (White arrow: fat replacement of subscapularis. Black arrow: atrophy of deltoid muscle. (B) Gluteal muscles (White arrow: fat replacement of gluteus medius and intermedius). (C) Mid‐thigh muscles show severe fat replacement except for rectus, femori, and sartorius. (D) Distal‐thigh muscles with less fat replacement, vastus muscles except for rectus femoris still affected. (E,F) Deep posterior compartment of calves is more affected proximally (arrow E) than distally (arrow F)

Full‐body T1‐weighted MRI along with dark fluid sequences of cerebrum was performed on a 3‐Tesla Siemens scanner (Magneton Verio Tim System, Erlangen, Germany). The T1‐w scan (Figure 2) showed significant fat replacement and atrophy of proximal muscle groups. In both the anterior and posterior compartments of the thigh several muscles were replaced by fat, with only rectus femoris, sartorius, gracilis, and biceps femoris being relatively preserved. Fat replacement and hypotrophy was less significant in the distal muscles of the calves, affecting only the deep posterior compartment and soleus muscle. In the shoulder girdle, subscapularis was replaced by fat and atrophy of the deltoid muscle was evident. Gluteus medius and intermedius of the pelvic girdle showed similar findings. Cerebral MRI (not shown) was normal and with no signs of hyperintensity in the putamen and no general cerebral atrophy. Two‐plan X‐ray of the spine showed straightened dorsal kyphosis and lumbar lordosis, but no scoliosis or gibbus deformity. In the lumbar vertebrae, skeletal changes were evident with reduced discus space and degenerative deposits. Two‐plan X‐ray of the hips showed no abnormalities (X‐rays not shown).

### 2.3 Biopsy

**FIGURE 3:**
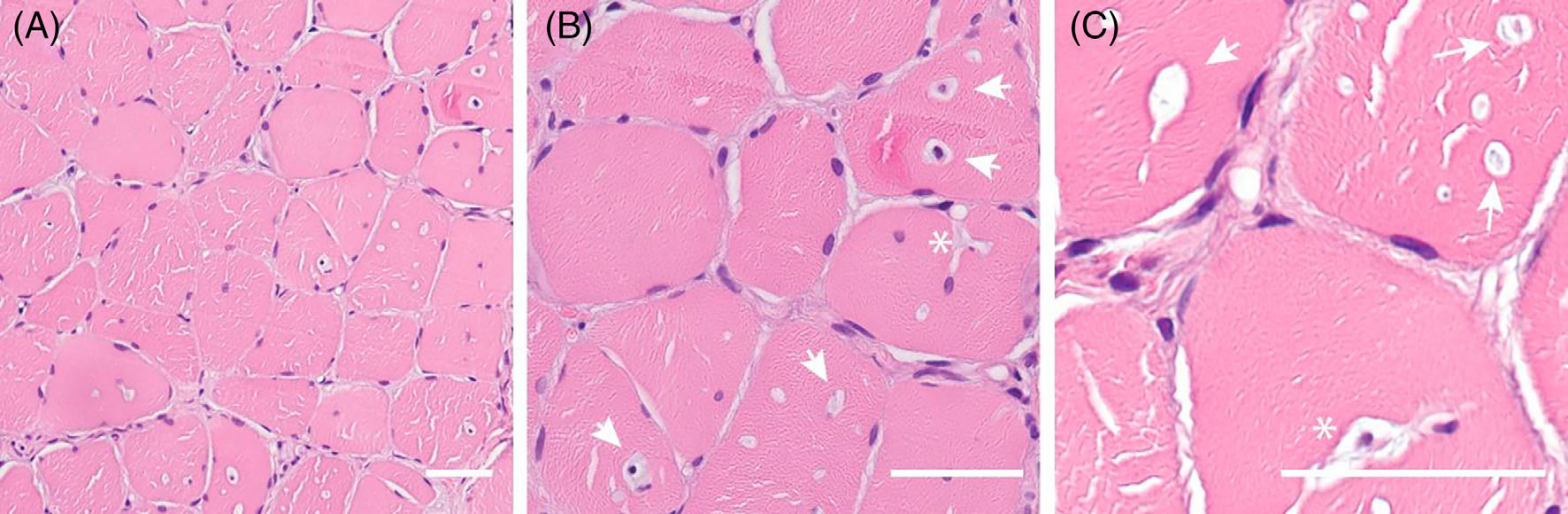
Histology of a vastus lateralis muscle biopsy demonstrates a mild myopathy with vacuoles and sarcolemmal invaginations. (A) HE stained section demonstrates mild myopathy and vacuoles with occasional central nuclei due to degeneration/regeneration cycle. (B,C) Vacuoles with or without nuclei (white arrows) are evident. Invaginations (white asterisk) are seen as well, occasionally with a nucleus inside the invagination. Bar is 50 μm

Muscle biopsy obtained at age 21 from the quadriceps femoris was sectioned and stained with hematoxylin and eosin for histopathological evaluation (Figure 3). Sections demonstrated mild to moderate myopathy with internal nuclei and cell infiltrations, indicative of a muscle disease. Heterogeneous vacuoles and sarcolemma invaginations were found scattered throughout the section. There were no signs of fibrofatty replacement, necrotic fibers, or morphological features suggesting congenital myopathy.

### 2.4 Metabolic analyzes

β‐galactosidase was analyzed twice by fluorescence enzyme assay with synthetic substrate, showing first 28% residual activity compared to 12 controls and secondly 34% activity compared to 16 controls. Quantitative 1,9‐dimethylmethylene dye‐binding test showed mildly elevated total urinary GAG (5.9–8.6 mg/mmol, ref.:0–5.1 mg/mmol) and qualitative analysis by mono‐dimensional electrophoresis indicated excess keratan sulfate secretion. Thin layer chromatography indicated normal urinary oligosaccharide secretion.

### 2.5 Additional laboratory findings

Biochemistry showed CK 194 U/L (ref: 35–210), ALAT 61 U/L (ref. 10–45 U/L), and LDH 300 U/L (ref. 105–205 U/L). Investigation by magnetic resonance cholangiopancreatography showed enlarged spleen. Echocardiography showed normal heart morphology and normal left ventricular ejection fraction. Forced volume capacity was reduced to 2.1 L, (52% of expected reference). Dry blood spot test for α‐glucosidase showed normal enzyme activity. Electroneurography showed normal motor conducting speed and amplitude in median nerve to adductor policis brevis muscle and in the peroneal nerve to extensor digitorum brevis. Sensory conduct ion speed and amplitudes in the same nerves and sural nerves were likewise normal. Electromyography showed myopathy in extensor digitorum communis sinister and few bursts of myotonia discharges in biceps brachii dexter and vastus medialis sinister.

## 3 DISCUSSIONS

In this case, we presented a patient with *GLB1*‐disorder accompanied by severe muscle wasting. The patient was followed in neuromuscular outpatient clinic for 20 years with progressive muscular involvement during which neuromuscular disorders were thoroughly investigated. A dry blood spot test for α‐glucosidase excluded Pompe disease, which was considered a relevant differential diagnosis due to respiratory compromise. The muscle biopsy presented signs of myopathy, but with no specific findings. The electromyography supported the presence of myopathy and normal electroneurography, together with electromyographic findings, excluded a neurogenic component. The clue for the diagnosis was given by the opportunity of exome sequencing, showing two *GLB1* variants. Clinical findings found in our patients, such as bone changes, cataract changes, splenomegaly, and respiratory compromise, are commonly described in patients with GM1 gangliosidosis.^6^ One study involving two Japanese patients with β‐galactosidase deficiency has previously described muscular atrophy in this patient group.^9^ We propose that earlier reports on *GLB1*‐related disorders have provided limited muscular assessments likely because of the predominant CNS and bone involvement usually representing the leading complications in these phenotypes especially when present at birth.^3^, ^7^

Residual enzyme activity of GLB1 was relatively high (28%–34%) compared to cases presenting at birth.^10^ Similar higher residual activity is, however, frequently reported in late onset cases.^7^, ^11^, ^12^, ^13^ Laboratory findings also supported a β‐galactosidase‐deficiency with increased total GAG and indication of keratan sulfate on electrophoresis. Although oligosaccharide accumulation on thin layer chromatography was absent, finding a specific oligosaccharide pattern only raises suspicion, but is not a diagnostic criterion.^1^ Collectively, the molecular genetics, biochemical and clinical findings all align well with other late onset cases of β‐galactosidase‐deficiency.^14^

GLB1‐related disorders compromise two phenotypes, GM1 and Morquio B. MRI findings such as hyperintensity of the putamen or general atrophy would have supported a GM1 phenotype,^15^ and spondyloepyseal dysplasia on the contrary would have supported a Morquio B phenotype.^3^ None of these specific findings was evident for this patient. While previously thought of as two distinct phenotypes, Morquio B and GM1 are increasingly being considered two disorders on a continuous spectrum with overlapping symptoms and metabolic findings.^7^, ^16^ In this case, we describe the patient as a late onset ß‐galactosidase‐deficiency patient with otherwise mild symptoms and but a severe muscular component. Even though the pathophysiological process in this case is still unknown to us, a muscular component in patient with lysosomal storage is not uncommon,^17^ and we propose that these findings may reflect a late onset symptom of β‐galactosidase‐deficiency.

The primary problem for our patient was the impaired lower limb weakness, impacting severely on walking, climbing stairs, and getting up from sitting. Diagnosis was delayed because of this predominant muscular presentation. This patient is the first case of *GLB1*‐related disorder to be described thoroughly through muscular assessment. Pathogenic *GLB1* variants should be considered in patients with unclassified muscle weakness and atrophy.

**Nomenclature**
GAG: glycosaminoglycan
MBD: morquio B disease

## AUTHOR CONTRIBUTIONS

Jonas Jalili Pedersen has made substantial contributions to conception and design, acquisition of data and analysis and interpretation of data. Morten Duno developed acquisition of data and analysis and interpretation of data. Flemming Wibrand developed acquisition of data and analysis and interpretation of data. Thomas Krag has been involved in drafting the manuscript and revising it critically for important intellectual content. Christian Hammer developed acquisition of data and analysis and interpretation of data. John Vissing has made substantial contributions to conception and design and has been involved in drafting the manuscript, revising it critically for important intellectual content.

## ETHICAL APPROVAL

All procedures followed were in accordance with the ethical standards of the responsible committee on human experimentation (institutional and national) and with the Helsinki Declaration of 1975, as revised in 2000 (5). Informed consent was obtained from all patients for being included in the study. We confirm that we have read JIMD guidelines on issues involved in ethical publication and affirm that this report is consistent with those guidelines.

## FUNDING INFORMATION

This research did not receive any specific grant from funding agencies in the public, commercial or not‐for‐profit sectors.

## CONFLICT OF INTEREST

Jonas Jalili Pedersen, Morten Duno, Flemming Wibrand, Christian Hammer, Thomas Krag, John Vissing declare that they have no conflict of interest.

## Data Availability

Most data supporting this study is presented in the result section. Patient specific data can be made available in anonymized form upon reasonable request, taking in regard GDPR regulations.
